# Prediction of endoscopic postoperative recurrence in Crohn’s disease: a nationwide multicenter real-world cohort from China

**DOI:** 10.1093/gastro/goag108

**Published:** 2026-10-07

**Authors:** Fan Hu, Zicheng Huang, Liangru Zhu, Qian Cao, Hong Guo, Yu Fu, Lingjie Huang, Lingya Xiang, Kang Chao, Chao Wang, Xiang Gao, Minhu Chen

**Affiliations:** Department of Gastroenterology, The First Affiliated Hospital, Sun Yat-sen University, No. 58 Zhongshan Er Road, Yuexiu District, Guangzhou, Guangdong 510062, P. R. China; Department of Gastroenterology, The Sixth Affiliated Hospital, Sun Yat-sen University, No. 26 Yuancun Erheng Road, Tianhe District, Guangzhou, Guangdong 510655, P. R. China; Guangdong Provincial Key Laboratory of Colorectal and Pelvic Floor Diseases, The Sixth Affiliated Hospital, Sun Yat-sen University, No. 26 Yuancun Erheng Road, Tianhe District, Guangzhou, Guangdong 510655, P. R. China; Biomedical Innovation Center, The Sixth Affiliated Hospital, Sun Yat-sen University, No. 7 Xingdao Huanbei Road, Haizhu District, Guangzhou, Guangdong 510320, P. R. China; Department of Gastroenterology, Union Hospital, Tongji Medical College, Huazhong University of Science and Technology, No. 1277 Jiefang Avenue, Jianghan District, Wuhan, Hubei 430022, P. R. China; Department of Gastroenterology, Sir Run Run Shaw Hospital, College of Medicine, Zhejiang University, No. 3 East Qingchun Road, Shangcheng District, Hangzhou, Zhejiang 310016, P. R. China; Inflammatory Bowel Disease Center, Sir Run Run Shaw Hospital, College of Medicine, Zhejiang University, No. 3 East Qingchun Road, Shangcheng District, Hangzhou, Zhejiang 310016, P. R. China; Department of Gastroenterology, Chongqing General Hospital, Chongqing University, No. 118 Xingguang Avenue, Liangjiang New Area, Chongqing 401147, P. R. China; Department of Gastroenterology, Union Hospital, Tongji Medical College, Huazhong University of Science and Technology, No. 1277 Jiefang Avenue, Jianghan District, Wuhan, Hubei 430022, P. R. China; Department of Gastroenterology, Sir Run Run Shaw Hospital, College of Medicine, Zhejiang University, No. 3 East Qingchun Road, Shangcheng District, Hangzhou, Zhejiang 310016, P. R. China; Inflammatory Bowel Disease Center, Sir Run Run Shaw Hospital, College of Medicine, Zhejiang University, No. 3 East Qingchun Road, Shangcheng District, Hangzhou, Zhejiang 310016, P. R. China; Department of Gastroenterology, Chongqing General Hospital, Chongqing University, No. 118 Xingguang Avenue, Liangjiang New Area, Chongqing 401147, P. R. China; Department of Gastroenterology, The Sixth Affiliated Hospital, Sun Yat-sen University, No. 26 Yuancun Erheng Road, Tianhe District, Guangzhou, Guangdong 510655, P. R. China; Guangdong Provincial Key Laboratory of Colorectal and Pelvic Floor Diseases, The Sixth Affiliated Hospital, Sun Yat-sen University, No. 26 Yuancun Erheng Road, Tianhe District, Guangzhou, Guangdong 510655, P. R. China; Biomedical Innovation Center, The Sixth Affiliated Hospital, Sun Yat-sen University, No. 7 Xingdao Huanbei Road, Haizhu District, Guangzhou, Guangdong 510320, P. R. China; Department of Pathology, The Sixth Affiliated Hospital, Sun Yat-sen University, No. 26 Yuancun Erheng Road, Tianhe District, Guangzhou, Guangdong 510655, P. R. China; Biomedical Innovation Center, The Sixth Affiliated Hospital, Sun Yat-sen University, No. 26 Yuancun Erheng Road, Tianhe District, Guangzhou, Guangdong 510655, P. R. China; Department of Gastroenterology, The Sixth Affiliated Hospital, Sun Yat-sen University, No. 26 Yuancun Erheng Road, Tianhe District, Guangzhou, Guangdong 510655, P. R. China; Guangdong Provincial Key Laboratory of Colorectal and Pelvic Floor Diseases, The Sixth Affiliated Hospital, Sun Yat-sen University, No. 26 Yuancun Erheng Road, Tianhe District, Guangzhou, Guangdong 510655, P. R. China; Biomedical Innovation Center, The Sixth Affiliated Hospital, Sun Yat-sen University, No. 7 Xingdao Huanbei Road, Haizhu District, Guangzhou, Guangdong 510320, P. R. China; Department of Gastroenterology, The First Affiliated Hospital, Sun Yat-sen University, No. 58 Zhongshan Er Road, Yuexiu District, Guangzhou, Guangdong 510062, P. R. China

**Keywords:** endoscopic recurrence, postoperative, Crohn’s disease, pathologic, prediction model

## Abstract

**Background:**

Preventing endoscopic postoperative recurrence (ePOR) remains a major challenge in Crohn’s disease (CD). This study aimed to identify the clinical and histopathological predictors of CD ePOR.

**Methods:**

This nationwide, multicenter retrospective cohort study included 389 CD patients from five tertiary Chinese IBD centers, all of whom underwent curative ileocolonic resection between July 2016 and June 2024. The primary endpoint was ePOR at 6–12 months after surgery, defined as a modified Rutgeerts’ score of ≥ i2b. Prediction models were developed based on the result of multivariate analysis of clinical and pathological data.

**Results:**

The overall ePOR rate was 24.9%. Multivariate analysis showed that prior exposure to biologics [odds ratio (OR) 2.695, 95% confidence interval (CI) 1.605–4.525, *P *< 0.001] and baseline serum albumin < 35 g/L (OR 2.095, 95% CI 1.160–3.785, *P *= 0.014) were independent risk factor for ePOR, while preoperative exclusive enteral nutrition (OR 0.338, 95% CI 0.188–0.610, *P *< 0.001) and early postoperative prophylactic biologic therapy (OR 0.503, 95% CI 0.304–0.832, *P *= 0.007) were independently associated with a reduced risk of ePOR. Moderate-to-severe lymphoplasmacytic infiltration in the lamina propria (OR 4.924, 95% CI 2.028–11.957, *P *< 0.001) and lymphangiectasia (OR 4.916, 95% CI 2.395–10.092, *P *< 0.001) were identified as the significant histopathological predictors at the surgical margins. A prediction model integrating both clinical and pathological variables [area under the receiver operating characteristic curve (AUROC) 0.829] significantly outperformed the clinical-only model (AUROC 0.715, *P *= 0.001).

**Conclusions:**

In the era of biologics and perioperative optimization, the 6–12-month ePOR rate among CD patients was 24.9%. A combined clinical–pathological model showed strong predictive performance.

## Introduction

Crohn’s disease (CD) is a chronic, non-specific inflammatory bowel disorder characterized by a relapsing-remitting course and progressive disease deterioration [1, 2]. Epidemiological data indicate that approximately 50% of patients underwent ileocolonic resection within 10 years of diagnosis prior to the biologic era [3]. With the advent of biologic therapies, surgical rates have declined to some extent, reflecting improved disease control [4–7]. However, surgery remains necessary in a substantial proportion of patients, particularly those with complications such as strictures or fistulas [8].

Importantly, surgery is not curative, as postoperative recurrence remains a major concern. Studies have shown that clinical recurrence occurs in 10%–38% of patients within the first year, while endoscopic recurrence during the same period has been reported in 35%–85% of cases [9]. In recent years, the widespread use of biologic agents, along with advances in preoperative optimization and early postoperative prophylaxis, has transformed perioperative management strategies [10, 11]. These changes highlight the need for updated real‑world data on endoscopic postoperative recurrence (ePOR)—defined as a modified Rutgeerts score of ≥ i2b, a threshold associated with higher risks of surgical and severe endoscopic recurrence [12]—to inform current clinical decision‑making.

The American Gastroenterological Association guidelines have outlined key risk factors for postoperative recurrence and emphasized the importance of accurate risk prediction and timely intervention [13]. However, most existing studies have focused on isolated clinical risk factors, and robust predictive models that integrate both clinical and histopathological variables are lacking [14, 15].

As the first large-scale, multicenter, retrospective real-world study in China, the present study aimed to evaluate the current rate of ePOR in CD patients 6–12 months after surgery under contemporary treatment paradigms. Furthermore, we sought to develop a predictive model incorporating both clinical and pathological variables to support individualized postoperative management.

## Methods

### Study design

It was a multicenter, retrospective, observational real-world cohort study. Patients were enrolled from five tertiary inflammatory bowel disease centers in China (the Sixth Affiliated Hospital of Sun Yat-sen University, Guangzhou, Guangdong; Sir Run Run Shaw Hospital of Zhejiang University School of Medicine, Hangzhou, Zhejiang; the First Affiliated Hospital, Sun Yat-sen University, Guangzhou, Guangdong; Union Hospital affiliated to Tongji Medical College, Huazhong University of Science and Technology, Wuhan, Hubei; Chongqing People’s Hospital, Chongqing). CD patients who underwent curative ileocolonic resection and had an endoscopic examination after surgery between July 2016 and June 2024 were considered for inclusion.

This study was approved by the Ethics Committee of the First Affiliated Hospital, Sun Yat-sen University (approval number [2024] No.293) and, thereafter, by all the other centers.

### Patient recruitment

Inclusion criteria were as follows: (i) adult patients (≥18 years old) with a confirmed diagnosis of CD according to current guidelines [16, 17]; (ii) patients who underwent curative ileocolonic resection of all macroscopic diseased bowel and ileocolonic anastomosis; (iii) patients who underwent endoscopic examination between 6 and 12 months after surgery.

Exclusion criteria were as follows: (i) colonoscopies that failed to reach the ileocolonic anastomosis or provided insufficient detail for assessing endoscopic disease activity or (ii) patients with a bowel stoma at the time of follow-up.

### Clinical data collection

Patients were routinely scheduled for endoscopic evaluation at 6–12 months postoperatively to monitor for disease recurrence. Baseline characteristics and follow‑up data were retrospectively retrieved from electronic medical records. The following variables were collected: sex, age, baseline smoking status, disease duration, clinical symptoms, history of appendectomy, disease location and behavior according to the Montreal classification, disease activity assessed by the CD Activity Index, medication history, C‑reactive protein and serum albumin (ALB) levels, preoperative therapy (within 4 weeks before surgery), early postoperative therapy (within 3 months after surgery), indication for surgery, type of surgical anastomosis, and postoperative endoscopic findings.

### Pathological characteristics of surgical margins

All hematoxylin and eosin-stained histology slides of the surgical resection specimen were centrally collected. Two experienced gastrointestinal pathologists independently and blindly evaluated the slides in a randomized order, following a standardized assessment protocol. Regular consensus meetings were held to define the histopathological features, resolve ambiguous cases, and reach consensus interpretations. The following features at the ileal resection margins were assessed on all available hematoxylin and eosin slides: presence of abscess, crypt abscess, ulceration, erosions, transmural inflammation, infiltration of neutrophils, lymphoplasmacytes, and eosinophils in the lamina propria, villous atrophy, pyloric gland metaplasia, lymphoid follicles, beaded lymphoid follicles, fibrous tissue hyperplasia, muscular layer hyperplasia, ganglion cell hyperplasia, nerve bundle hyperplasia, plexitis, granuloma, lymphangiectasia, and vasculitis. Interobserver agreement was evaluated for each histological feature using Cohen’s kappa statistics.

### Outcomes

The primary endpoint was the occurrence of ePOR, defined as a modified Rutgeerts’ score of ≥ i2b on ileocolonoscopy performed 6–12 months after surgery. The secondary endpoint was to identify clinical and pathological predictors of ePOR and to construct predictive models based on these factors. Model 1 included clinical variables only, whereas Model 2 incorporated both clinical and pathological variables. The predictive performance of the two models was compared.

### Statistical analysis

All statistical analyses were performed using R software (version 4.4.2, R Foundation for Statistical Computing, Vienna, Austria) and SPSS (version 26.0, IBM Corp., Armonk, NY, USA). Continuous variables were expressed as mean ± standard deviation for normally distributed data or median with interquartile range for non-normally distributed data. Categorical variables were expressed as counts and percentages. All statistical tests were two-sided, and a *P* value < 0.05 was considered statistically significant.

For variable selection, all candidate variables were first subjected to univariate logistic regression analysis. Variables with a *P* value < 0.1 were then included in a multivariate logistic regression model to identify independent predictors. Based on the results of multivariate analysis, two prediction models were constructed: Model 1 included clinical variables only, while Model 2 incorporated both clinical and pathological variables. Model performance was evaluated using receiver operating characteristic (ROC) curves, calibration plots, and decision curve analysis to assess discrimination, calibration, and clinical utility, respectively. Internal validation was performed via bootstrap resampling (*B* = 1,000). The areas under the ROC curves (AUCs) were compared using DeLong’s test. A nomogram was also developed based on the final regression model to facilitate individualized risk estimation and clinical application.

## Results

### Study population

A total of 427 patients who received ileocolonic resection between July 2016 and June 2024 were considered for inclusion, of whom 389 met the inclusion criteria and were enrolled in the study (Fig. 1). In the overall cohort (Cohort 1), the median disease duration was 56.3 (29.9, 101.1) months, and 33.7% of the patients had a prior history of biologic therapy. Preoperative exclusive enteral nutrition (EEN) for at least 4 weeks was administered in 34.2% of patients, while prophylactic biologic therapy within 3 months after surgery was initiated in 53.5%. The most common surgical indications were intestinal stricture (77.1%) and fistulas (58.6%). Among the 389 patients, 204 (52.4%) had complete pathological data available from the ileal resection margins and were classified as the pathological subgroup (Cohort 2). Interobserver agreement for histopathological assessments was substantial, with a Cohen’s kappa coefficient of 0.74 [95% confidence interval (CI): 0.69–0.78] (Supplementary Table S1). Baseline clinical characteristics and histopathological features are summarized in Tables 1 and 2, respectively.

**Figure 1:**
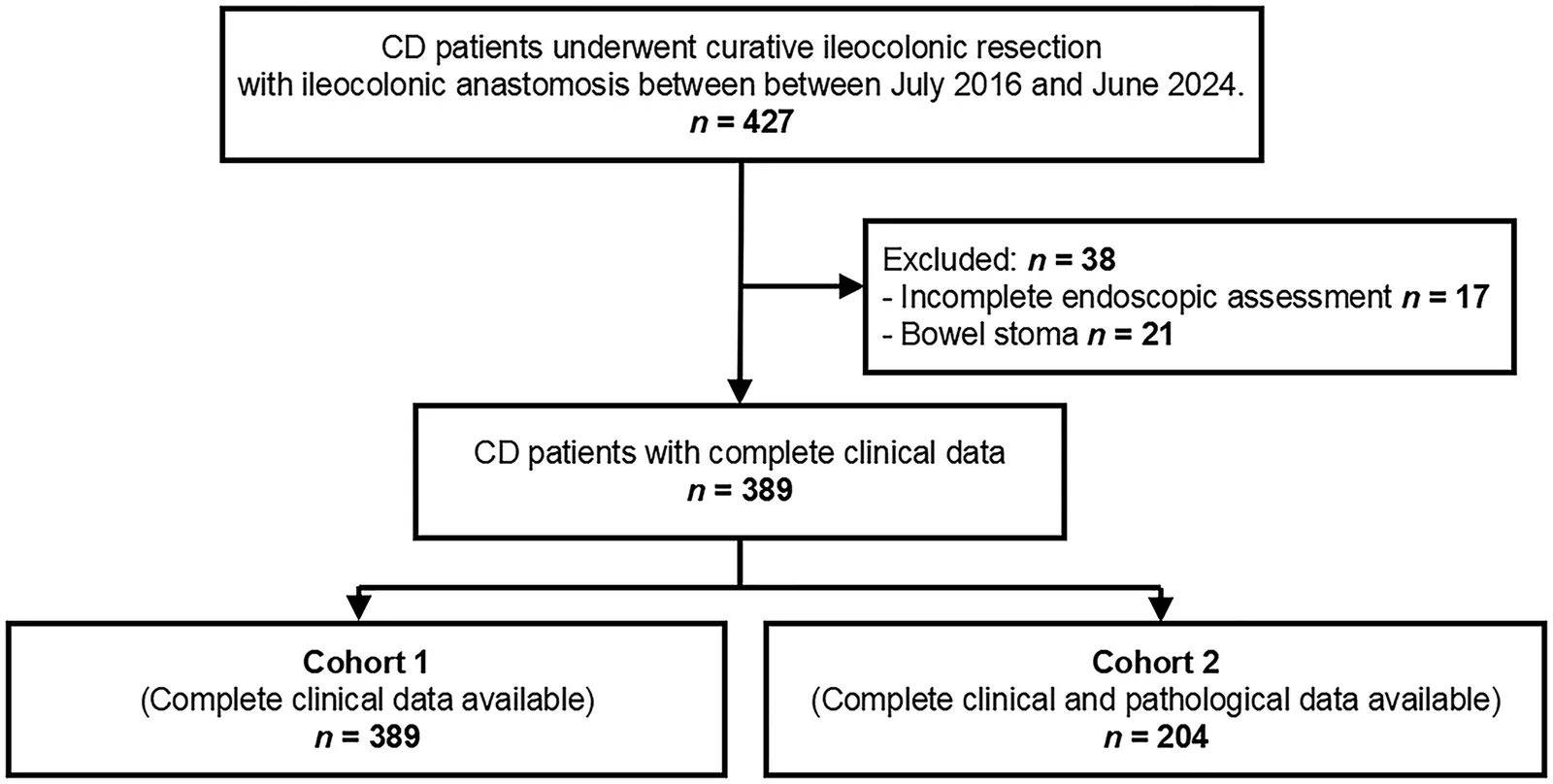
Flowchart illustrating the selection of patients in this study. CD, Crohn’s disease.

**Table 1:** Baseline clinical characteristics of included patients

Characteristic	Total (Cohort 1, *n *= 389)	Subgroup (Cohort 2, *n *= 204)
Male	268 (68.9)	131 (64.2)
Age, years	30.0 (24.4, 38.0)	30.5 (24.3, 39.0)
Disease duration, months	56.3 (29.9, 101.1)	50.4 (20.9, 98.4)
Active smokers	17 (4.4)	17 (8.3)
Previous appendectomy	49 (12.6)	27 (13.2)
Location		
L1	51 (13.1)	17 (8.3)
L2	12 (3.1)	4 (2.0)
L3	326 (83.8)	183 (89.7)
L4	136 (35.0)	93 (45.6)
Disease behavior		
B1	38 (9.8)	17 (8.3)
B2	118 (30.3)	52 (25.5)
B3	233 (59.9)	135 (66.2)
Perianal disease	188 (48.3)	105 (51.5)
Bio-exposed	131 (33.7)	83 (40.7)
Previous steroid therapy	91 (23.4)	53 (26.0)
Previous immunomodulator therapy	120 (30.8)	75 (36.8)
Preoperative biologics	40 (10.3)	6 (2.9)
Preoperative exclusive enteral nutrition	133 (34.2)	68 (33.3)
Crohn’s Disease Activity Index at baseline	216.9 (112.5, 306.3)	162.5 (75.0, 262.5)
Surgical indications		
Stenosis	300 (77.1)	171 (83.8)
Fistula	228 (58.6)	136 (66.7)
Perforated	12 (3.1)	9 (4.4)
Hemorrhage	7 (1.8)	5 (2.5)
Others	24 (6.2)	3 (1.5)
Emergency surgery	30 (7.7)	13 (6.4)
Phase II coincidence	124 (31.9)	73 (35.8)
Anastomotic method		
End-to-end anastomosis	27 (6.9)	7 (3.4)
Side-to-side anastomosis	303 (77.9)	183 (89.7)
End-to-side anastomosis	59 (15.2)	15 (7.4)
Early postoperative biologics therapy	208 (53.5)	102 (50.0)
Early postoperative steroid or immunomodulator therapy	68 (17.5)	37 (18.1)
C‑reactive protein, mg/L	3.1 (0.9, 16.7)	1.8 (0.6, 8.4)
C‑reactive protein > 10 mg/L	130 (33.4)	43 (21.1)
Serum albumin, g/L	39.8 (36.0, 42.9)	40.4 (38.2, 42.9)
Serum albumin < 35 g/L	78 (20.1)	21 (10.3)

The values are presented as numbers (percentages) or medians (interquartile ranges).

**Table 2:** Baseline pathological characteristics of included patients

Characteristic	*n *= 204
Abscess	13 (6.4)
Crypt abscess	1 (0.5)
Ulcer	5 (2.5)
Erosion	53 (26.0)
Lamina propria neutrophilia	
None-mild	198 (97.1)
Moderate-severe	6 (2.9)
Lamina propria lymphoplasmacytic hyperplasia	
None-mild	175 (85.8)
Moderate-severe	29 (14.2)
Lamina propria eosinophilia	
None-mild	185 (90.7)
Moderate-severe	19 (9.3)
Villus atrophy	75 (36.8)
Pyloric gland metaplasia	8 (3.9)
Lymphoid follicle	
0	110 (53.9)
1	48 (23.5)
2–3	27 (13.2)
> 3	19 (9.3)
Transmural inflammation	2 (1.0)
Beaded lymphoid follicles	22 (10.8)
Fibrous tissue hyperplasia	111 (54.4)
Muscular layer hyperplasia	24 (11.8)
Ganglion cell hyperplasia	24 (11.8)
Nerve bundle hyperplasia	43 (21.1)
Plexitis	9 (4.4)
Granuloma	5 (2.5)
Lymphangiectasia	64 (31.4)
Vasculitis	28 (13.7)

The values are presented as numbers (percentages) or medians (interquartile ranges).

### Endoscopic recurrence

The overall rate of ePOR at 6–12 months was 24.9% (97 of 389). Among these, Rutgeerts scores of i2b, i3, and i4 accounted for 17.7%, 2.3%, and 4.9%, respectively (Fig. 2A).

**Figure 2:**
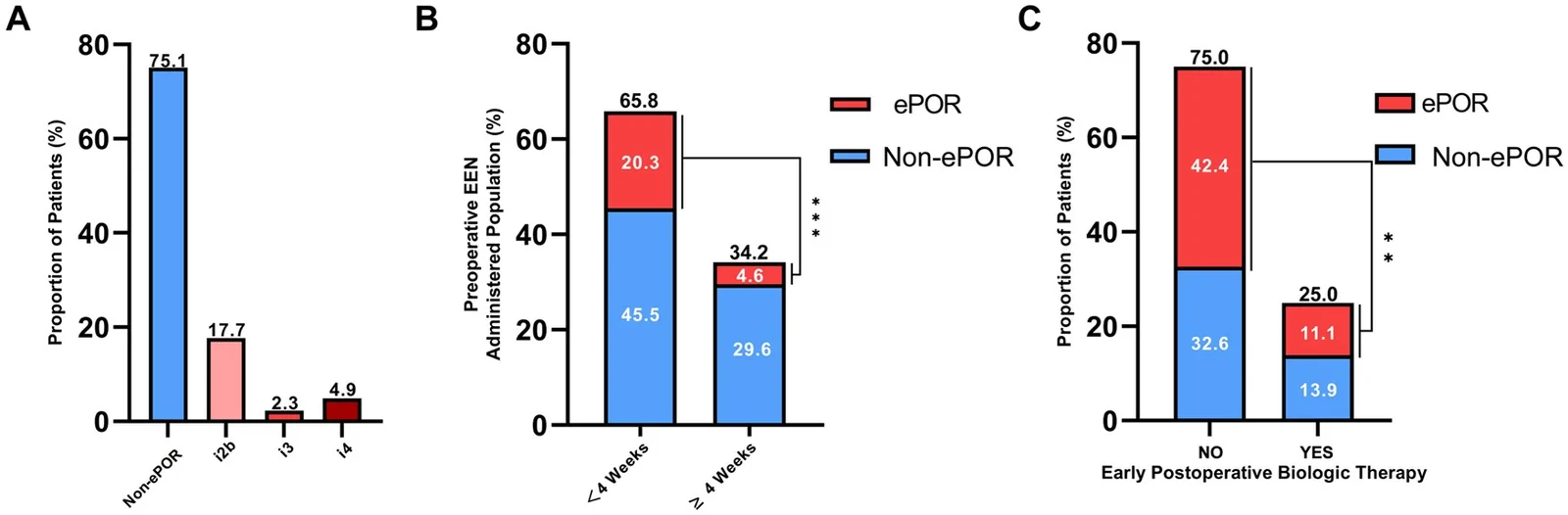
Proportion of ePOR in CD and its association with preoperative EEN and early postoperative biologic therapy in Cohort 1. (A) Endoscopic recurrence at 6–12 months after surgery. (B) The ePOR rate of patients with different preoperative EEN history. (C) The ePOR rate of patients with or without early postoperative biologics therapy. **, *P *< 0.01; ***, *P *< 0.001; ePOR, endoscopic postoperative recurrence; EEN, exclusive enteral nutrition.

### Risk factors for postoperative recurrence

Clinical variables were analyzed in Cohort 1. Univariate analysis identified several variables associated with ePOR at a *P* value < 0.1, including age, disease duration, prior exposure to biologics, prior steroid use, preoperative EEN for at least 4 weeks, baseline ALB < 35 g/L, and early postoperative biologic therapy. These variables were subsequently entered into a multivariate logistic regression model. Multivariate analysis revealed that prior exposure to biologics [odds ratio (OR) 2.695, 95% CI 1.605–4.525, *P *< 0.001] and baseline ALB < 35 g/L (OR 2.095, 95% CI 1.160–3.785, *P *= 0.014) were independent risk factors for ePOR. In contrast, preoperative EEN (OR 0.338, 95% CI 0.188–0.610, *P *< 0.001) (Fig. 2B) and early postoperative prophylactic biologic therapy (OR 0.503, 95% CI 0.304–0.832, *P *= 0.007) (Fig. 2C) were independently associated with a reduced risk of ePOR (Supplementary Table S2).

Pathological variables were identified in Cohort 2, which included patients with complete pathological data. Univariate analysis revealed that moderate-to-severe lymphoplasmacytic infiltration in the lamina propria and lymphangiectasia at the ileal resection margin were associated with a higher risk of ePOR (*P *< 0.1). Multivariate analysis confirmed that both moderate-to-severe lymphoplasmacytic infiltration (OR 4.924, 95% CI 2.028–11.957, *P *< 0.001) and lymphangiectasia (OR 4.916, 95% CI 2.395–10.092, *P *< 0.001) were independent pathological predictors of ePOR (Supplementary Table S3).

**Table 3:** Performance of predictive models for endoscopic recurrence

Prediction model	AUROC (95% CI)	Bootstrap-corrected AUROC (optimism)	Sensitivity	Specificity	PPV	NPV
Model 1^a^	0.715 (0.636–0.793)	0.692 (0.026)	56.3%	75.0%	40.9%	84.8%
Model 2^b^	0.829 (0.761–0.897)	0.803 (0.027)	77.1%	79.5%	53.6%	91.9%

The probability of developing an endoscopic recurrence can be estimated using the following predictive formula. Coefficients in the final prediction model were adjusted for optimism using shrinkage factors derived from internal validation. For each variable, a value of 1 was assigned if the condition was present and 0 if absent.

a The logistic regression Model 1 was expressed as follows:

logit(P) = −1.143 + 0.972 × bioexposed − 1.164 × preoperative EEN + 0.990 × ALB < 35 g/L − 0.647 × early postoperative biologics.

b The logistic regression Model 2 was expressed as follows:

logit(P) = −1.993 + 0.836 × bioexposed − 1.163 preoperative EEN + 0.918 × ALB < 35 g/L − 0.554 × early postoperative biologics + 1.425 × moderate-to-severe lymphoplasmacytic infiltration + 1.561 × lymphangiectasia.

EEN, exclusive enteral nutrition; AUROC, area under the receiver operating characteristic curve; CI, confidence interval; PPV, positive predictive value; NPV, negative predictive value; ALB, serum albumin.

### Construction of predictive models for postoperative endoscopic recurrence

Based on the results of the multivariate logistic regression analyses, predictive models were constructed using data from Cohort 2, which included patients with complete clinical and pathological information. Model 1 incorporated only clinical variables, whereas Model 2 integrated both clinical and pathological variables. The AUROC for model 1 was 0.715 (95% CI 0.636–0.793). After incorporating pathological variables, the AUROC of Model 2 increased to 0.829 (95% CI 0.761–0.897), indicating a significantly improved predictive performance (DeLong’s test, *P *= 0.001). Furthermore, decision curve analysis indicated that Model 2 demonstrated superior clinical utility compared with Model 1, with a higher net benefit across a wide range of threshold probabilities (Fig. 3A and B).

**Figure 3:**
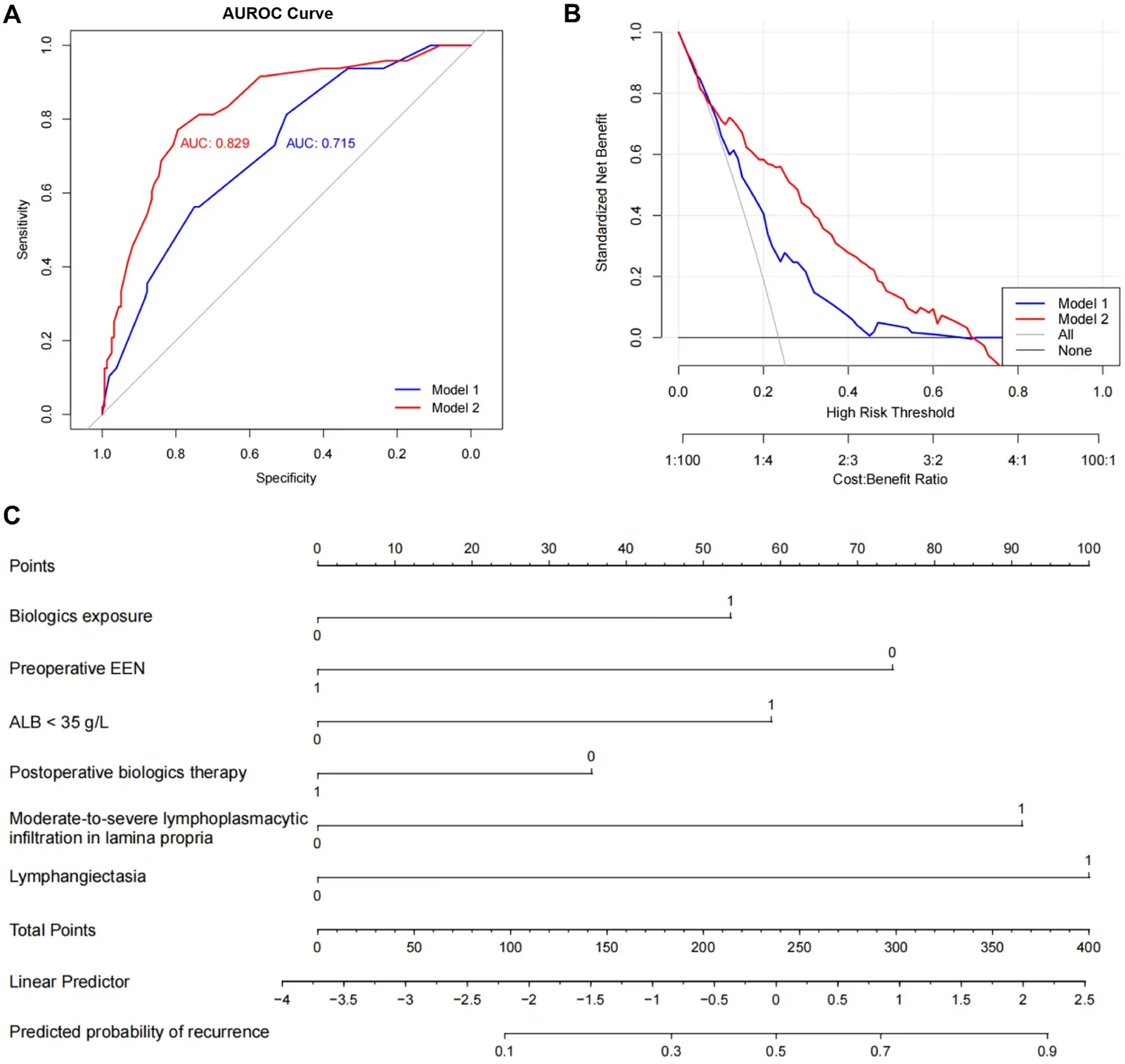
Performance evaluation of Model 1 and Model 2. (A) The AUROC curves of both models. (B) The decision curve analysis of both models. (C) The visualization of model 2 as a nomogram. ROC, receiver operating characteristic; AUROC, the areas under the ROC curve; AUC, the areas under the ROC curve; ALB, serum albumin; EEN, exclusive enteral nutrition.

Internal validation using bootstrap resampling (*B* = 1,000) demonstrated calibration slopes (shrinkage coefficients) of 0.862 for Model 1 and 0.855 for Model 2, indicating minimal overfitting. The optimism-corrected AUROCs were 0.692 (optimism = 0.026) for Model 1 and 0.803 (optimism = 0.027) for model 2, suggesting that both models maintained satisfactory discriminative ability after correction, with low optimism bias (Table 3). Calibration curves are presented in Supplementary Fig. S1. A nomogram based on Model 2 is illustrated in Fig. 3C to facilitate individualized risk estimation.

## Discussion

In this nationwide, multicenter, real-world study, we found that the rate of ePOR within 6 to 12 months after surgery was 24.9%. Additionally, we developed a predictive model integrating both clinical and pathological variables, which demonstrated good performance in estimating endoscopic recurrence risk.

Previous studies have reported markedly higher rates of ePOR following CD surgery. Two meta-analyses indicated that mucosal lesions developed in up to 70% of patients within the first year after bowel resection, with approximately 50% occurring near the anastomosis [18, 19]. A randomized controlled trial also demonstrated that, among patients without early endoscopic surveillance at 6 months after surgery, the rate of ePOR (defined as Rutgeerts score ≥ i2) reached 67% at 18 months following standard medical therapy [20]. In contrast, the recurrence rate observed in our cohort was substantially lower, at approximately 25%, which may reflect the benefits of improved perioperative strategies, including nutritional optimization and early postoperative prophylactic biologic use. The relatively high recurrence rates observed in earlier studies may be attributed to differences in therapeutic strategies, as our study was conducted in the context of recent advances in medical therapy and evolving approaches to perioperative management. In our cohort, more than 30% of patients received preoperative EEN, and nearly 50% initiated prophylactic biologic therapy early after surgery.

The European Crohn’s and Colitis Organisation guidelines recommend comprehensive preoperative assessment and risk-stratified prophylactic therapy for CD patients undergoing elective surgery [8], and our findings align with this guidance. Multivariate analysis confirmed that both preoperative EEN and early postoperative biologic therapy were independently associated with reduced risk of ePOR. A prospective cohort study reported that at least 4 weeks of preoperative EEN improved inflammatory and nutritional status, thereby reducing both postoperative complications following laparoscopic surgery and the rate of endoscopic recurrence during follow-up [21]. Another retrospective study indicated that biologic therapy was significantly associated with a reduced risk of ePOR (OR 0.22, 95% CI 0.1–0.51), as well as better long-term outcomes, including lower rates of hospitalization and reoperation [22]. Due to the limitations of the retrospective study design, we were unable to compare the efficacy of different biologic agents in preventing postoperative recurrence. Nevertheless, multiple studies have demonstrated that anti-TNF-α agents and newer biologics, such as vedolizumab and ustekinumab, effectively reduce the risk of ePOR [23–26]. These findings support that early initiation of preoperative prophylactic biologic therapy may provide more effective control of intestinal inflammation and subsequently lower the risk of ePOR [27].

In terms of clinical predictors, we identified prior biologic exposure and preoperative hypoalbuminemia as independent risk factors for ePOR. Prior biologic exposure may reflect suboptimal disease control before surgery, indicating a higher inflammatory burden that predisposes patients to postoperative recurrence. In addition, hypoalbuminemia indicates poor nutritional status and more active disease, highlighting the importance of preoperative optimization [28].

The histopathological features at the surgical margins provide valuable microscopic insights into the risk of ePOR. In our study, two significant predictors were identified. Moderate-to-severe lymphoplasmacytic infiltration in the lamina propria likely represents ongoing microscopic inflammation that is often undetectable during intraoperative visual inspection and may therefore serve as a more sensitive histological marker. A systematic meta-analysis confirmed that positive histological margins—including lymphoplasmacytic infiltration—were associated with increased risks of both clinical and endoscopic recurrence (risk ratio 1.87, 95% CI 1.14–3.08, *I*² = 71%) [16]. Another key pathological feature, lymphangiectasia, may reflect a persistently activated subclinical inflammatory state and is frequently accompanied by lamina propria infiltration. Lymphangiectasia is commonly observed in chronic inflammatory diseases, including inflammatory bowel disease, and may lead to tissue edema, lymphocytopenia, hypoproteinemia, and broader immune dysregulation [29, 30]. Mechanistically, such lymphatic remodeling may indicate impaired drainage and retention of immune cells, thereby promoting a pro-inflammatory microenvironment conducive to disease recurrence [31].

To enhance clinical applicability, we developed a predictive model and constructed a nomogram to enable individualized risk estimation. Our findings demonstrated that incorporating pathological variables alongside clinical parameters significantly improved the model’s predictive performance for ePOR compared with using clinical variables alone. This suggested that pathological features offer additional prognostic value beyond conventional clinical factors. Whereas clinical variables predominantly reflect macroscopic disease burden and treatment history, pathological indicators provide insight into underlying histological activity and the biological behavior of the disease. This multidimensional integration of clinical and pathological data not only improves recurrence risk stratification but also deepens our understanding of disease mechanisms, thereby laying a foundation for personalized postoperative preventive strategies.

This study has several notable strengths. First, to our knowledge, it represents the first large-scale, multicenter, real-world cohort in China to investigate ePOR in CD and uniquely identifies predictive factors from both clinical and histopathological perspectives. Second, we developed a multidimensional prediction model integrating clinical and pathological variables. This comprehensive approach to identifying high-risk patients for postoperative recurrence offers valuable evidence to support early, proactive postoperative interventions in CD management. However, several limitations should also be acknowledged. First, as a retrospective real-world study, it is subject to inherent biases associated with retrospective data collection. Second, due to limitations in sample size and data completeness, some potentially relevant variables could not be analyzed, which may have affected the findings. Third, the prediction model underwent internal validation only. Although it demonstrated good performance with minimal overfitting, external validation in independent prospective cohorts is warranted to confirm its generalizability.

## Conclusions

Under current treatment paradigms—including widespread biologic use and optimized perioperative strategies—the 6–12-month ePOR rate in CD patients was 24.9%. The prediction model integrating both clinical and pathological variables exhibited good discriminatory performance. However, its clinical applicability requires further validation in large, independent prospective cohorts.

## Supplementary Material

goag108_Supplementary_Data
