# Supplementary material for: Prediction of endoscopic postoperative recurrence in Crohn’s disease: a nationwide multicenter real-world cohort from China
Source: Gastroenterol Rep (Oxf). 2026 Oct 7;14:goag108. doi: 10.1093/gastro/goag108 (PMC13644528; doi:10.1093/gastro/goag108)
Supplement: Supplementary file 1 [file goag108_supplementary_data.zip › Supplementary_materials_final version 20260810.docx]

**Supplementary materials**

**Supplementary Table 1.** Interobserver agreement between pathologists.

| Characteristic | Cohen's Kappa κ |
| --- | --- |
| Abscess | 0.78 |
| Crypt abscess | 0.66 |
| Ulcer | 0.85 |
| Erosion | 0.72 |
| Lamina propria neutrophilia | 0.60 |
| Lamina propria lymphoplasmacytic hyperplasia | 0.68 |
| Lamina propria eosinophilia | 0.70 |
| Villus atrophy | 0.66 |
| Pyloric gland metaplasia | 0.73 |
| Lymphoid follicle | 0.66 |
| Transmural inflammation | 0.78 |
| Beaded lymphoid follicles | 0.80 |
| Fibrous tissue hyperplasia | 0.75 |
| Muscular layer hyperplasia | 0.83 |
| Ganglion cell hyperplasia | 0.82 |
| Nerve bundle hyperplasia | 0.75 |
| Plexitis | 0.73 |
| Granuloma | 0.75 |
| Lymphangiectasia | 0.76 |
| Vasculitis | 0.70 |

**Supplementary Table 2.** Clinical predictive factors analysis for endoscopic postoperative recurrence at 6–12 months.

| Variable | Univariate analysis | | |  | Multivariate analysis | | |
| --- | --- | --- | --- | --- | --- | --- | --- |
|  | Odds ratio | 95% confidence interval | *P* value |  | Odds ratio | 95% confidence interval | *P* value |
| Age | 1.020 | 0.999–1.041 | 0.062 |  | 1.016 | 0.994–1.039 | 0.152 |
| Disease duration | 1.005 | 1.001–1.009 | 0.016 |  | 1.003 | 0.998–1.007 | 0.252 |
| Crohn’s Disease Activity Index > 150 | 1.075 | 0.662–1.773 | 0.771 |  |  |  |  |
| Female | 1.195 | 0.728–1.940 | 0.474 |  |  |  |  |
| Smoke | 1.268 | 0.395–3.518 | 0.663 |  |  |  |  |
| Previous appendectomy | 0.746 | 0.340–1.503 | 0.434 |  |  |  |  |
| Location |  |  |  |  |  |  |  |
| L2 vs L1 | 0.780 | 0.383–1.588 | 0.493 |  |  |  |  |
| L3 vs L2 | 0.258 | 0.033–2.026 | 0.198 |  |  |  |  |
| L4 | 0.837 | 0.508–1.355 | 0.474 |  |  |  |  |
| Behavior |  |  |  |  |  |  |  |
| B2 vs B1 | 0.666 | 0.279–1.592 | 0.361 |  |  |  |  |
| B3 vs B1 | 1.051 | 0.634–1.742 | 0.847 |  |  |  |  |
| Perianal disease | 1.007 | 0.635–1.595 | 0.978 |  |  |  |  |
| Bioexposed | 2.197 | 1.370–3.525 | 0.001 |  | 2.695 | 1.605–4.525 | **< 0.001** |
| Previous steroid use | 1.582 | 0.935–2.640 | 0.082 |  | 1.401 | 0.791–2.482 | 0.248 |
| Previous immunosuppressant | 1.215 | 0.740–1.972 | 0.435 |  |  |  |  |
| Preoperative biologics | 1.160 | 0.535–2.360 | 0.692 |  |  |  |  |
| Preoperative exclusive enteral nutrition | 0.351 | 0.195–0.604 | < 0.001 |  | 0.338 | 0.188–0.610 | **< 0.001** |
| C‑reactive protein > 10 mg/L | 0.975 | 0.593–1.579 | 0.918 |  |  |  |  |
| Serum albumin < 35 g/L | 1.823 | 1.056–3.102 | 0.026 |  | 2.095 | 1.160–3.785 | **0.014** |
| Surgical indications |  |  |  |  |  |  |  |
| Stenosis | 0.808 | 0.479–1.394 | 0.434 |  |  |  |  |
| Fistula | 1.197 | 0.750–1.928 | 0.454 |  |  |  |  |
| Perforated | 1.004 | 0.219–3.444 | 0.996 |  |  |  |  |
| Hemorrhage | 1.208 | 0.171–5.707 | 0.823 |  |  |  |  |
| Emergency surgery | 1.051 | 0.428–2.344 | 0.907 |  |  |  |  |
| Anastomotic method |  |  |  |  |  |  |  |
| Side to side vs end to end | 1.848 | 0.709–4.818 | 0.209 |  |  |  |  |
| End-to-side vs end to end | 0.807 | 0.429–1.521 | 0.508 |  |  |  |  |
| Phase II coincidence | 1.139 | 0.694–1.846 | 0.601 |  |  |  |  |
| Postoperative biological therapy | 0.613 | 0.385–0.972 | 0.038 |  | 0.503 | 0.304–0.832 | **0.007** |
| Postoperative steroid or immunosuppressant therapy | 0.825 | 0.429–1.510 | 0.547 |  |  |  |  |

**Supplementary Table 3.** Pathological predictive factors analysis for endoscopic postoperative recurrence at 6–12 months.

| Variable | Univariate analysis | | |  | Multivariate analysis | | |
| --- | --- | --- | --- | --- | --- | --- | --- |
|  | Odds ratio | 95% confidence interval | *P* value |  | Odds ratio | 95% confidence interval | *P* value |
| Abscess | 0.573 | 0.123–2.681 | 0.479 |  |  |  |  |
| Crypt abscess | 0 | (0, -) | 1.000 |  |  |  |  |
| Ulcer | 2.217 | 0.360–13.676 | 0.391 |  |  |  |  |
| Erosion | 0.935 | 0.444–1.968 | 0.859 |  |  |  |  |
| Moderate-to-severe lamina propria neutrophilia | 0.643 | 0.073–5.638 | 0.690 |  |  |  |  |
| Moderate-to-severe lamina propria lymphoplasmacytic hyperplasia | 5.500 | 2.408–12.565 | < 0.001 |  | 4.924 | 2.028–11.957 | < 0.001 |
| Moderate-to-severe lamina propria eosinophilia | 1.179 | 0.402–3.461 | 0.764 |  |  |  |  |
| Lymphoid follicle | 0.882 | 0.630–1.236 | 0.466 |  |  |  |  |
| Villus atrophy | 1.312 | 0.677–2.541 | 0.421 |  |  |  |  |
| Pyloric gland metaplasia | 2.013 | 0.463–8.753 | 0.351 |  |  |  |  |
| Transmural inflammation | - | (0, -) | 0.999 |  |  |  |  |
| Beaded lymphoid follicles | 0.951 | 0.331–2.728 | 0.925 |  |  |  |  |
| Fibrous tissue hyperplasia | 1.377 | 0.713–2.661 | 0.340 |  |  |  |  |
| Muscular layer hyperplasia | 1.095 | 0.408–2.937 | 0.857 |  |  |  |  |
| Ganglion cell hyperplasia | 0.618 | 0.200–1.906 | 0.403 |  |  |  |  |
| Nerve bundle hyperplasia | 0.828 | 0.365–1.877 | 0.651 |  |  |  |  |
| Plexitis | 2.745 | 0.707–10.665 | 0.145 |  |  |  |  |
| Granuloma | 2.217 | 0.360–13.676 | 0.391 |  |  |  |  |
| Lymphangiectasia | 5.277 | 2.647–10.521 | < 0.001 |  | 4.916 | 2.395–10.092 | < 0.001 |
| Vasculitis | 1.098 | 0.436–2.765 | 0.844 |  |  |  |  |

**Supplementary Figure 1.** The calibration curves of (A) model 1 and (B) model 2.
